# 纳米纤维在线固相萃取检测尿液中3种儿茶酚胺和5-羟色胺

**DOI:** 10.3724/SP.J.1123.2021.07001

**Published:** 2021-12-08

**Authors:** Yueling BI, Tong XU, Liqin CHEN

**Affiliations:** 1.天津市西青医院药剂科, 天津 300380; 1. Department of Pharmacy, Tianjin Xiqing Hospital, Tianjin 300380, China; 2.天津医科大学公共卫生学院卫生毒理与卫生化学教研室, 天津 300070; 2. Department of Toxicology and Sanitary Chemistry, School of Public Health, Tianjin Medical University, Tianjin 300070, China; 3.天津市环境营养与人群健康重点实验室, 天津 300070; 3. Tianjin Key Laboratory of Environment, Nutrition and Public Health, Tianjin 300070, China

**Keywords:** 聚冠醚复合纳米纤维, 高效液相色谱, 在线固相萃取, 儿茶酚胺, 5-羟色胺, 尿, polymeric crown ether composite nanofiber, high performance liquid chromatography (HPLC), on-line solid phase extraction, catecholamines, serotonin, urine

## Abstract

生物单胺包括儿茶酚胺类以及5-羟色胺等,在中枢神经系统中扮演着非常关键的角色,也是临床上诊断神经内分泌肿瘤疾病的重要生物标志物。由于这类单胺类物质的强化学极性导致传统吸附材料对其吸附效果不佳,从复杂生物样本中同时检测更多的生物单胺存在挑战性。该文建立了一种基于聚冠醚纳米纤维在线固相萃取检测尿液中3种儿茶酚胺(多巴胺、肾上腺素、去甲肾上腺素)和5-羟色胺的方法。采用静电纺丝法制备聚二苯并-18-冠-6醚-聚苯乙烯复合纳米纤维(PCE-PS),制成装填纤维的固相萃取(PFSPE)柱,再将PFSPE柱与HPLC进行在线联用。该在线PFSPE-HPLC方法采用双三元泵进行样品富集净化和分析,左泵连接PFSPE柱,进行样品富集净化;右泵连接分析柱进行样品分离检测。控制切换阀的切换,实现样品富集后洗脱至分析柱中分离检测。结果表明,在线PFSPE-HPLC检测尿液儿茶酚胺(多巴胺、肾上腺素、去甲肾上腺素)和5-羟色胺在1~200 ng/mL范围内有良好的线性关系,线性相关系数达0.996以上。3种儿茶酚胺和5-羟色胺的检出限(*S/N*=3)分别为1和2.5 ng/mL,定量限(*S/N*=10)分别为2.5和5 ng/mL。空白尿液和实际尿液加标回收率在83.5%~117.7%之间,日内精密度<10%。PCE-PS复合纳米纤维在多次使用后无明显变化,具有良好的稳定性,可重复使用达95次以上。在线PFSPE-HPLC方法能够集样品在线前处理与分析检测于一体,省时省力,实现分析过程的高度自动化。该方法成功应用于尿液中3种儿茶酚胺和5-羟色胺的检测,可以为临床上相关疾病检测诊断和研究提供有力的技术支持。

## Abstract (en)

Biogenic monoamines, including catecholamines (CAs) and serotonin (5-HT), play critical roles in the central nervous system. They have recently been proven to be primarily useful as biomarkers for the diagnosis of CA-producing tumors. The highly polar properties of biogenic monoamines result in poor retention on conventional materials, making it challenging to simultaneously measure more biogenic monoamines from complex matrices. Moreover, the classical method of off-line pretreatment is relatively complex, labor-intensive, and incurs errors in repeatability among different operators. Therefore, the development of an on-line sample pretreatment method combined with the use of specific nanofiber adsorbents has been explored. An on-line procedure could avoid unnecessary and time-consuming steps, and enable full automation of the experimental process. In this study, an on-line packed-fiber solid-phase extraction (PFSPE) and determination method for urinary CAs (dopamine (DA), norepinephrine (NE), epinephrine (E)) and 5-HT was developed, using composite nanofibers of polycrown ether-polystyrene (PCE-PS). PCE-PS composite nanofibers prepared by electrospinning were used as adsorbents in the PFSPE column, which was connected to the on-line HPLC system. The PFSPE-HPLC equipment contained a dual ternary pump and a switching valve to enable enrichment, purification, and analysis directly in the system. The left pump was connected with the PFSPE column for sample enrichment and purification, while the right pump was attached to the analysis column for sample separation and testing. The switching valve was controlled such that after enrichment, the samples could be eluted to the analysis column for separation and detection.

The current work expands on our previous research by analyzing more target substances, and developing an on-line sample pretreatment method to simultaneously analyze four biogenic monoamines. Gradient separation aided in the satisfactory separation of the biogenic monoamines within a short retention time. The running time was set at 16 min to enable thorough enrichment, elution, and analysis. The influence of the complexing reagent (diphenylborinic acid 2-aminoethyl ester, 2 mg/mL) was also investigated with this on-line PFSPE-HPLC system. The results showed that the intensity of most analytes was significantly higher when 50 μL of the complexing reagent was added. The influence of a buffer on the extraction of the biogenic monoamines was also tested. The optimum extraction condition for the target analytes was achieved when artificial urine (AU) samples were diluted in a volume ratio of 1:1 by phosphate- buffered saline solution (PBS, pH 7.8). Under the optimum experimental conditions, the on-line PFSPE-HPLC procedure showed good linearity (in the range of 1 ng/mL to 200 ng/mL) with correlation coefficients above 0.996 for the quantitative detection of urinary CAs (DA, NE, E) and 5-HT. For the CAs, the limit of detection (LOD) was 1 ng/mL (*S/N*=3), while the limit of quantitation (LOQ) was 2.5 ng/mL (*S/N*=10). For 5-HT, the LOD was 2.5 ng/mL (*S/N*=3) and the LOQ was 5 ng/mL (*S/N*=10). Moreover, high recovery rates and good reproducibility were obtained. The recoveries of AU and real urine spiked with CAs and 5-HT were in the range of 83.5%-117.7%, and the intra-day precision was lower than 10%. Additionally, no significant changes in the nanofibers were observed after repeated extraction, which reflected the good stability and reusability of the nanofibers. The nanofibers could be reused for more than 95 times.

The on-line PFSPE-HPLC system was successfully applied for the determination of urinary CAs and 5-HT with good precision and high sensitivity. This high level of integration and automation was significantly advantageous in terms of its repeatability, as well as reduction in the time and effort required. The proposed on-line pretreatment and determination method can provide strong technical support for the detection and diagnosis of, as well as research on related diseases in clinical practice.

神经内分泌肿瘤是一类伴随着儿茶酚胺类(CAs)物质大量分泌的疾病^[1]^。由于儿茶酚胺类物质在人体内各种生理活动和免疫系统的调节方面起到非常重要的作用,所以这类物质除了能作为诊断神经内分泌肿瘤疾病的生物标志物外,还与多种神经系统和自身免疫性疾病有关,例如阿尔茨海默病、抑郁症、注意力缺陷多动障碍(ADHD)、精神分裂症、帕金森病、焦虑和类风湿关节炎等^[2,3,4]^。因此,生物样本中这些分析物的测定对于支持疾病监测和新的治疗药物开发具有重要的临床意义。然而,生物样本中儿茶酚胺类物质含量极低,且其他内源性物质干扰测定,所以生物样本中的此类痕量物质分析往往面临着巨大的困难,目标物信号常常要么达不到仪器的检出限,要么淹没在复杂浩瀚的干扰物质信号当中。

儿茶酚胺类物质主要包括肾上腺素(E)、去甲肾上腺素(NE)和多巴胺(DA)。另一种神经递质5-羟色胺(5-HT)也是近年来研究中使用较多的一类指标^[5,6]^。这一类物质化学极性强,在通常的C18等疏水性的固相介质上几乎无保留,所以说这类物质的前处理是极具挑战性的一类工作。而传统的此类物质样品前处理方法一般都采用离线操作的方式进行,耗时费力且易造成损耗误差,导致样品前处理方法成为整个样本分析流程的瓶颈问题^[7]^。而在线样品前处理与高效色谱检测方法联用能够为解决此瓶颈问题提供方法和思路,此联用方法不仅可以减轻技术人员的劳动强度,实现分析过程高度自动化,节约分析成本;更主要的是可以减少甚至消除由于手工操作中个体差异所产生的误差,提高分析测试的灵敏度、准确度与重现性^[8]^。再者,在线样品前处理和检测联用方式更容易实现试剂无毒化操作,减少对操作人员及环境的危害,从而促进绿色化学的发展。在线样品前处理与高效色谱检测方法联用在具备多种检测分析优势的基础上,还能提高分析样品的检测通量,近年来在样本分析领域得到了广泛的发展应用^[9,10]^。

基于电纺纳米纤维固相萃取(packed-fiber solid phase extraction, PFSPE)的技术正在蓬勃发展,其核心是以纳米纤维取代通行的颗粒状微米级固相吸附材料进行样品前处理。其可以针对待捕集目标分子的理化性质,用高压电纺技术纺制与之有选择性相互作用的纳米纤维,从而为建立极性分子的在线固相萃取分析平台奠定基础^[11,12]^。本文在已有研究基础^[13]^上,将PFSPE技术引入在线样品前处理体系,并拓宽分析目标物质,开发在线样品前处理方法。本研究只用普通的HPLC-FLD系统外加一个切换阀就可以同时实现NE、E、DA以及5-HT的在线前处理与分离检测,不仅能为神经内分泌肿瘤标志物的准确、灵敏检测提供更为高效的在线样品前处理检测体系,还可以拓宽在线样品前处理技术的应用范围。

## 1 实验部分

### 1.1 仪器和试剂

UltiMate3000双三元高效液相色谱仪、FLD-3100荧光检测器、Chromeleon 7.2 SR5色谱数据系统(美国Thermo Scientific公司)。DW-P403-1AC高压电源(天津东文高压电源厂),微量注射泵WZ-50C6(浙江史密斯医学仪器有限公司), 1-14小型台式离心机(美国Sigma-Aldrich公司), Milli-Q Integral超纯水系统(法国密理博公司)。

NE、E、DA、5-HT、3,4-二羟基苯乙胺氢溴酸(DHBA)、二苯硼酸2-氨基乙基酯(DPBA)均购自美国Sigma-Aldrich公司。甲醇、乙腈、冰乙酸(色谱纯,天津市康科德科技有限公司),聚苯乙烯(PS,相对分子质量1.8×10^5^,上海化学试剂研究所),二苯并-18-冠-6醚(天津希恩思生化科技有限公司)。二苯并-18-冠-6醚聚合物树脂冠醚(PCE)由天津医科大学卫生化学实验室合成提供。

### 1.2 标准样品制备和尿液样本处理

混合标准溶液:分别配制加超纯水溶解的1.0 mg/mL的NE、E、DA以及5-HT的母液(E标准品需先用30 μL 0.1 mol/L盐酸溶解),再吸取各母液适量,充分混匀配制成100 μg/mL的混合标准溶液,密封、避免光照、于冰箱-20 ℃下保存备用。

内标(IS)溶液:取内标物质DHBA适量,加入超纯水溶解,配制成1.0 mg/mL的IS储备液,密封、避免光照、于冰箱中-20 ℃下保存备用。

DPBA溶液:取DPBA适量加水配制成2.0 mg/mL溶液以备后用。

磷酸盐缓冲液(PBS):先配制0.2 mol/L的NaH_2_PO_3_水溶液和Na_2_HPO_3_水溶液,二者按61∶39、81∶19、183∶17、947∶53的体积比分别混合,对应缓冲液的pH分别为7.0、7.4、7.8和8.0。

人工尿液(AU):参考文献^[14]^中的配比进行人工尿液的配制,尿酸的加入量参考文献^[15]^,得到尿酸溶解完全的人工尿液。

尿液样本:新鲜收集的尿液采用0.22 μm的水膜过滤,再加入等体积的PBS(pH 7.8)与之混匀。

### 1.3 聚冠醚复合纳米纤维的制备

参考文献^[11]^制备聚合冠醚复合纳米纤维。取3.6 g(0.01 mol)二苯并-18-冠-6醚溶于20 mL甲酸溶液中;在三口烧瓶中加入0.6 g多聚甲醛,加入20 mL甲酸,加热(50 ℃)搅拌使其溶解,然后将配好的20 mL二苯并-18-冠-6醚的甲酸溶液经恒压漏斗缓慢滴加到三口烧瓶中,保持50 ℃继续搅拌4~5 h,观察有絮状物析出,室温下继续搅拌约20 h,有棕灰色固体沉淀产生,抽滤固体,用30 mL蒸馏水反复洗涤2~3次,置于送风干燥箱中彻底烘干,得到固体产品即为聚合冠醚。

静电纺丝过程如下:配制5%(v/v)PCE的二甲基亚砜(DMSO)溶液和15%(v/v)聚苯乙烯(PS)的二甲基甲酰胺-四氢呋喃(DMF-THF, 4∶6, v/v)溶液,将两种溶液按照4∶10(v/v)混匀作为纺丝前体溶液。将该溶液装入玻璃注射器中,其不锈钢针头与高压电源的阳极相连,铝箔收集设备与高压电源的阴极相连,二者距离为15 cm,电压为20 kV,进液速率为2.0 mL/h, PCE-PS溶液在高压电场下喷射形成复合纳米纤维。

### 1.4 PFSPE柱的制备

采用不锈钢细棒(直径为0.5 mm)将适量PCE-PS复合纳米纤维约10 mg分次填充于金属柱筒(10 mm×2.1 mm)中,压紧填实,装上两端筛板后放入外套管中组装。将组装好的PFSPE柱与UltiMate3000高效液相色谱仪相连接。

### 1.5 PFSPE-HPLC在线联用程序

采用仪器自带的双位十通切换阀设计了PFSPE-HPLC在线联用的程序,该程序分为3部分:(1)样品富集过程(见图1a),样品经自动进样器注入PFSPE柱中进行富集净化;(2)样品洗脱转移过程(见图1b),通过切换十通阀的阀位至2-1,样品被洗脱液洗脱,转移到分析柱中;(3)样品分析过程(见图1a),阀位再次切换回10-1,在分析柱中进行目标物的分离和检测,同时PFSPE柱再平衡,预备下针进样。程序的总运行时间为16.00 min。

**图1:**
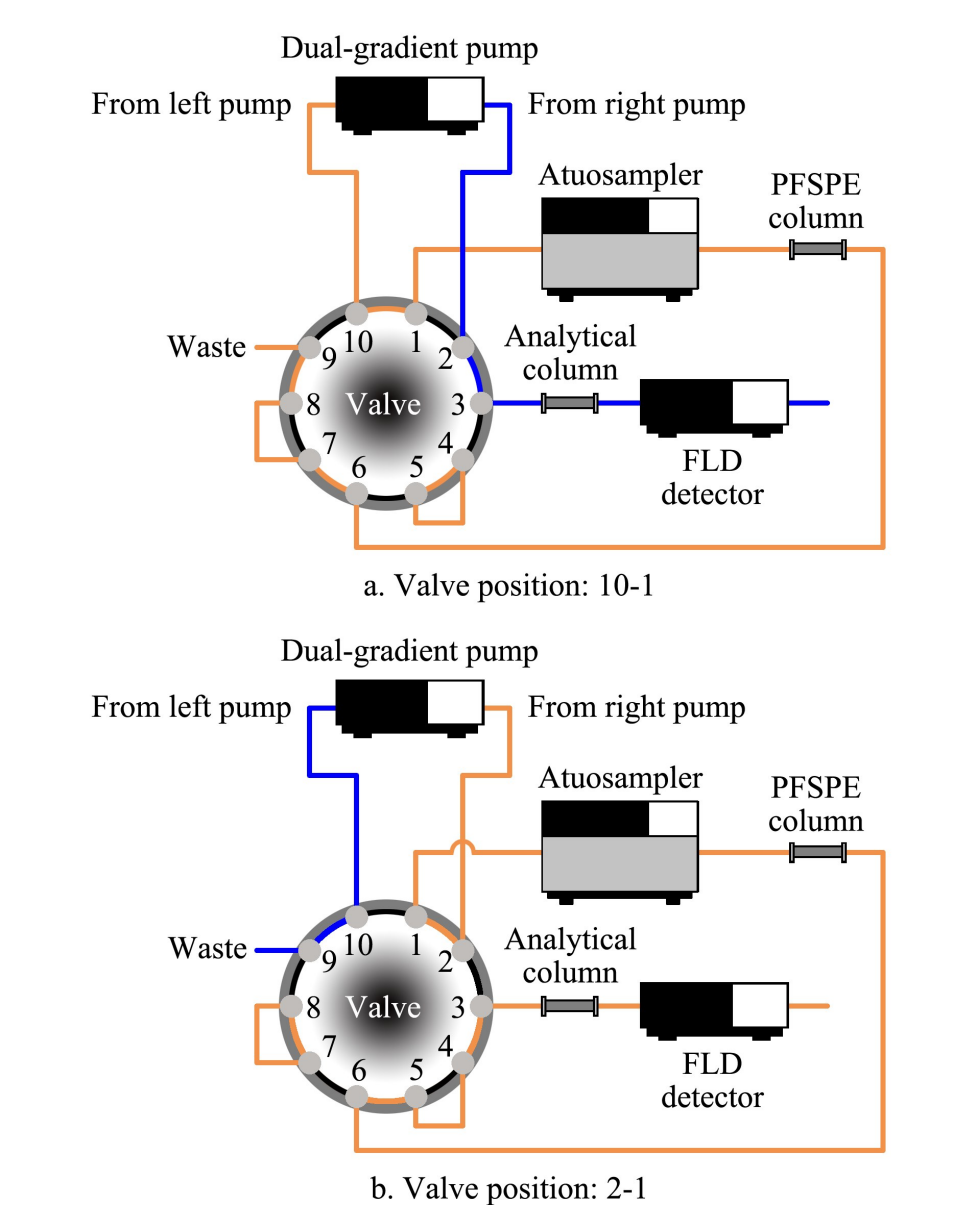
PFSPE-HPLC在线联用程序设计示意图

### 1.6 色谱条件

色谱柱选用YMC-Pack pro C18色谱柱(100 mm×3.0 mm, 5 μm);柱温设为35 ℃;荧光检测器的激发波长(*λ*_ex_)设为286 nm,发射波长(*λ*_em_)设为318 nm;流动相A:准确称量0.45 g庚烷磺酸钠、7.6 g柠檬酸、7.3 g磷酸二氢钠、0.1 g EDTA、1.9 g氢氧化钠于烧杯中,再加入55 mL乙腈,用超纯水定容至1 L,充分混匀,抽滤,超声脱气10 min,此时溶液的pH为4.15;流动相B: 1 mol/L冰醋酸;流速设为0.5 mL/min;进样量为100 μL。最佳梯度洗脱时间程序方法见表1。

**表1:** 最佳梯度洗脱时间程序方法

Time/ min	Flow rate/ (mL/min)	Left pump (enrichment) mobile phase (water)/%	Right pump (analysis)		Valve position
			Mobile phase A/%	Mobile phase B/%	
0.0	0.5	100	100	0	10-1
3.0	0.5	100	100	0	10-1
3.5	0.5	100	0	100	2-1
4.5	0.5	100	0	100	10-1
5.0	0.5	100	15	85	10-1
15.0	0.5	100	60	40	10-1
16.0	0.5	100	100	0	10-1

### 1.7 标准曲线的绘制

采用人工尿液配制1、5、10、20、50、100、200 ng/mL的系列标准混合溶液(其中IS的质量浓度均为25 ng/mL),依次注入PFSPE-HPLC在线联用系统中,测定各个质量浓度下的各目标物峰面积。以质量浓度*X*(ng/mL)为横坐标,各目标物与IS的峰面积比值*Y*为纵坐标,分别绘制NE、E、DA和5-HT的标准曲线。

### 1.8 加标回收率试验

采用人工尿液加标以及健康人尿液测试基底值后加标进行加标回收率试验。

尿液基质:吸取0.5 mL人工尿液或实际尿液,按照体积比1∶1加入pH 7.8的PBS后,再加入2.5 μg/mL的IS溶液10 μL和2.0 mg/mL DPBA溶液50 μL,混匀。

人工尿液加标分为低、中、高3个水平(10、50、100 ng/mL)。吸取0.5 mL人工尿液,按照体积比1∶1加入pH 7.8的PBS后,分别加入10 μL混合标准溶液(1、5、10 μg/mL),再加入2.5 μg/mL的IS溶液10 μL和2.0 mg/mL DPBA溶液50 μL,混匀。

实际尿液加标100 ng/mL。吸取0.5 mL尿液,按照体积比1∶1加入pH 7.8的PBS后,加入10 μL混合标准溶液(10 μg/mL),再加入2.5 μg/mL的IS溶液10 μL和2.0 mg/mL DPBA溶液50 μL,混匀。

分别取人工尿液和实际尿液基质及其加标溶液各100 μL进样分析,记录各峰面积。计算加标回收率:加标回收率=(测定值-基质本底值)/加标水平×100%。

## 2 结果与讨论

### 2.1 PFSPE-HPLC在线联用程序的优化

PFSPE-HPLC在线联用程序的重要操作参数是流速、流动相梯度,以及富集、转移时间、分析和平衡时间。如果富集时间过短,会导致目标物质没有吸附充分,过长会导致不必要的运行时间。转移时间过短会导致目标物质没有充分转移。分析时间过短则会造成目标物质不能全部出峰,过长也会导致运行时间过长。净化平衡时间过短也会造成PFSPE柱和分析柱未平衡完全。此外,色谱柱的填料、粒度、内径及柱长等都会影响其柱效,进而影响分离度及灵敏度等。因此,本实验对以上影响因素都进行了优化,最终优化的条件见1.5、1.6节,NE、E、DA、5-HT和IS的分离色谱图如图2所示,在此优化条件下,杂质峰较少,基线较为平稳,分离较充分,有利于准确的定性定量分析。

**图2:**
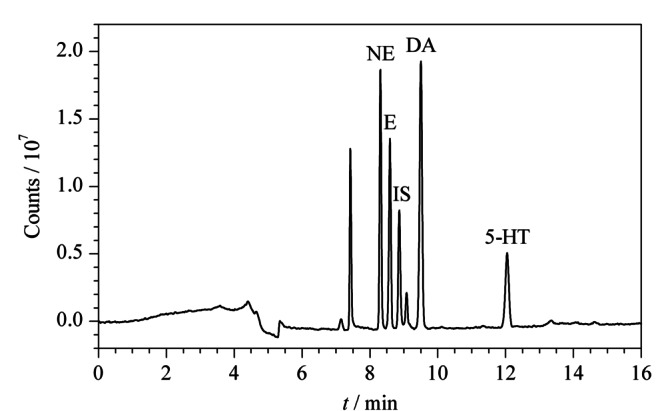
最佳梯度洗脱时间程序方法下的100 ng/mL NE、E、DA、5-HT和IS标准物质混合水标液分离色谱图

### 2.2 络合剂DPBA加入量的影响

由于DPBA和儿茶酚胺基团之间存在可逆的络合作用^[5,16]^,而络合反应产物可以提高在PFSPE柱上的吸附效率,所以本试验测试了DPBA加入量对分析物的检测影响。采用人工尿液配制加标溶液1 mL(NE、E、DA和5-HT的质量浓度为100 ng/mL, IS为25 ng/mL),加入不同体积的DPBA(2 mg/mL)。从图3可知,DPBA的加入对于CAs的作用非常明显,而对5-HT的作用有限。总体而言,加入量为50 μL的条件下,各目标物质的峰面积最高,说明此条件为DPBA的最佳加入量。因此后续实验都采取此条件,加入50 μL DPBA。

**图3:**
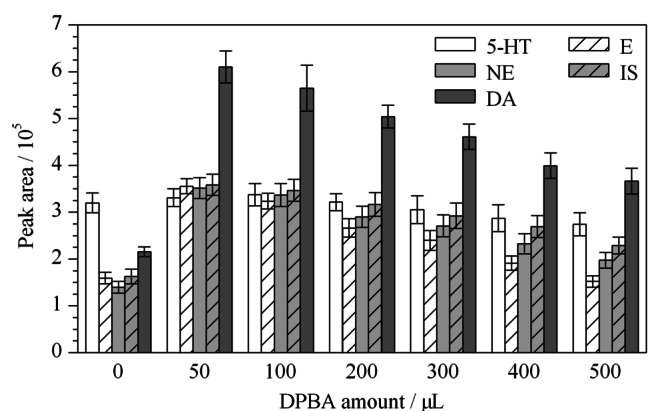
络合试剂DPBA(2 mg/mL)加入量对分析物提取效果的影响(*n*=3)

### 2.3 PBS的影响

在普通膳食条件下,正常人的尿液偏酸性,pH均值保持在6.3左右^[17]^。由于DPBA络合实验需要在近中性条件下反应为佳,所以采用PBS对尿液pH进行调节。本实验测试了不同pH的PBS以及人工尿液与不同pH的PBS等体积混合溶液中的各分析物响应值。如图4所示,在单纯PBS中,pH 7.0条件下各分析物的峰响应值最大。而在人工尿液与PBS等体积混合的溶液中,pH 7.8条件下各分析物的峰响应值最佳。此结果也说明采用一定量的PBS调节人工尿液的pH至近中性后,各分析物能够得到较好的提取。

**图4:**
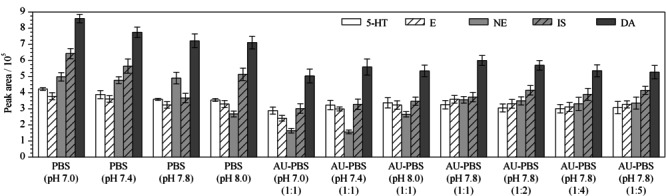
PBS对分析物检测的影响(*n*=3)

此外,在4种体积比条件下混合人工尿液和pH 7.8的PBS,各分析物的峰响应值差异较小,1∶1条件下各分析物的峰响应值稍好于其他3种配比条件,说明此条件已达到最佳吸附pH条件,所以在后续的实验中人工尿液或实际尿液的pH调节都采用pH 7.8的PBS等体积比混合。

### 2.4 PFSPE柱的稳定性及重复使用次数

前期实验中发现,PFSPE柱经上百次使用后,纳米纤维只有略微的机械变形,直径无显著变化,纳米结构保持完整,可以重复使用^[15]^。本实验对PFSPE柱的重复使用次数进行了统计,以进一步考察PFSPE柱的稳定性。实验结果发现,该PFSPE柱在PFSPE-HPLC系统中可以进行近200次CAs和5-HT的在线自动化富集和分析。取1.2节配制的混合标准溶液加超纯水稀释至100 ng/mL(IS质量浓度为25 ng/mL)进样100 μL, NE、E、DA、5-HT及IS的峰面积及NE、E、DA、5-HT与IS的峰面积比随使用次数的变化见图5(统计到95次),图中显示出NE、E、DA及IS的峰面积均随使用次数的增加先下降后上升,然后缓慢下降到一定水平,再在该水平的基础上有长时间的小幅波动,最后又继续下降。而5-HT的峰面积能够在较长时间内保持较好的稳定性(5-HT和CAs类化学结构有差异,所以变化趋势有可能不一致)。这些都说明PFSPE柱具有一定的重复使用性和较好的稳定性,可以在一定时间内保持稳定,建议使用过程中随时采用标准溶液进行质控,以保持对PFSPE性能的了解,确保标准溶液和实际尿液的测试条件尽量在较短时间内保持一致,减少因PFSPE前处理柱的性能因素而导致的实验误差。

**图5:**
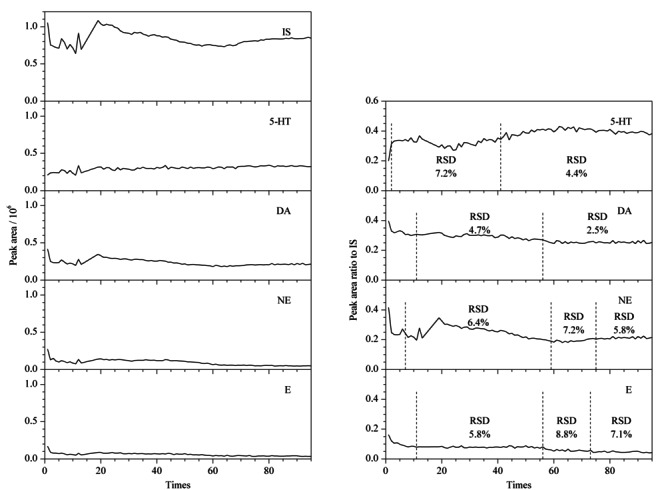
PFSPE柱的重复使用次数对分析物检测的影响(*n*=95)

### 2.5 方法学

2.5.1 标准曲线

本实验采用内标法定量。在1~200 ng/mL的范围内,NE、E、DA、5-HT的色谱峰清晰可见。以NE、E、DA、5-HT与IS的色谱峰面积比值(*Y*)对各单胺的质量浓度(*X*)做线性回归,结果显示NE、E和DA在1~200 ng/mL之间呈线性,5-HT在5~200 ng/mL之间呈线性,且线性关系良好(相关系数*r* ≥0.996)。检出限为1 ng/mL (*S/N*=3, CAs)和2.5 ng/mL (*S/N*=3, 5-HT),定量限为2.5 ng/mL (*S/N*=10, CAs)和5 ng/mL (*S/N*=10, 5-HT)。NE、E、DA、5-HT的线性方程分别为:*Y*=0.0089*X*+0.0335 (NE, *r*=0.9993), *Y*=0.0073*X*+0.0558(E, *r*=0.9961), *Y*=0.0136*X*+0.0042(DA, *r*=0.9997), *Y*=0.0052*X*+0.0030(5-HT, *r*=0.9998)。

2.5.2 加标回收率

人工尿液和实际尿液加标回收试验结果见表2, NE、E、DA及5-HT的加标回收率分别为87.0%~117.7%、87.6%~110.7%、83.5%~110.0%及98.7%~111.7%,相对标准偏差(RSD)均小于10%。回收率和精密度都符合测试要求,说明该检测方法适用于尿液样本中NE、E、DA及5-HT的定量分析。

**表2:** 人工尿液和实际尿液样品的加标回收率(*n*=6)

Compound	Recoveries of artificial urine (RSDs)/%				Actual urine		
	10 ng/mL	50 ng/mL	100 ng/mL		Background/(ng/mL)	Spiked/(ng/mL)	Recovery (RSD)/%
NE	87.0 (4.2)	102.5 (6.5)	108.3 (5.3)		15.5	100.0	117.7 (3.6)
E	87.6 (6.6)	105.9 (5.3)	105.6 (7.1)		3.7	100.0	110.7 (8.6)
DA	83.5 (7.0)	88.0 (9.9)	110.0 (7.8)		170.6	100.0	106.7 (4.8)
5-HT	98.7 (8.4)	111.7 (7.9)	104.9 (7.8)		53.2	100.0	101.6 (9.8)

### 2.6 实际尿液样本的检测

取健康人的随机尿中段尿1份,按照1.8小节项下的操作配成尿液基质,进样100 μL,计算尿液基质中NE、E、DA及5-HT对IS的峰面积比值,将峰面积比值代入到各分析物的标准曲线中求出尿液中NE、E、DA及5-HT的质量浓度。

由图6可知,实际尿液样本中的NE、E、DA、5-HT经PFSPE柱在线富集和净化后能够在HPLC-FLD系统中得到较好的分离与检出,峰形优良,与杂质分离效果好,便于NE、E、DA及5-HT的定性定量分析。该方法中,样品在线富集净化及检测所需的时间在16 min内,方便快捷、大大节省人力物力。实际尿液样本中NE、E、DA和5-HT的质量浓度分别为100.0、45.9、311.7和197.8 ng/mL。3种CAs和5-HT的含量范围与文献^[18]^报道中的数值范围基本吻合,说明此方法能够应用于测定尿液中的NE、E、DA和5-HT的含量。

**图6:**
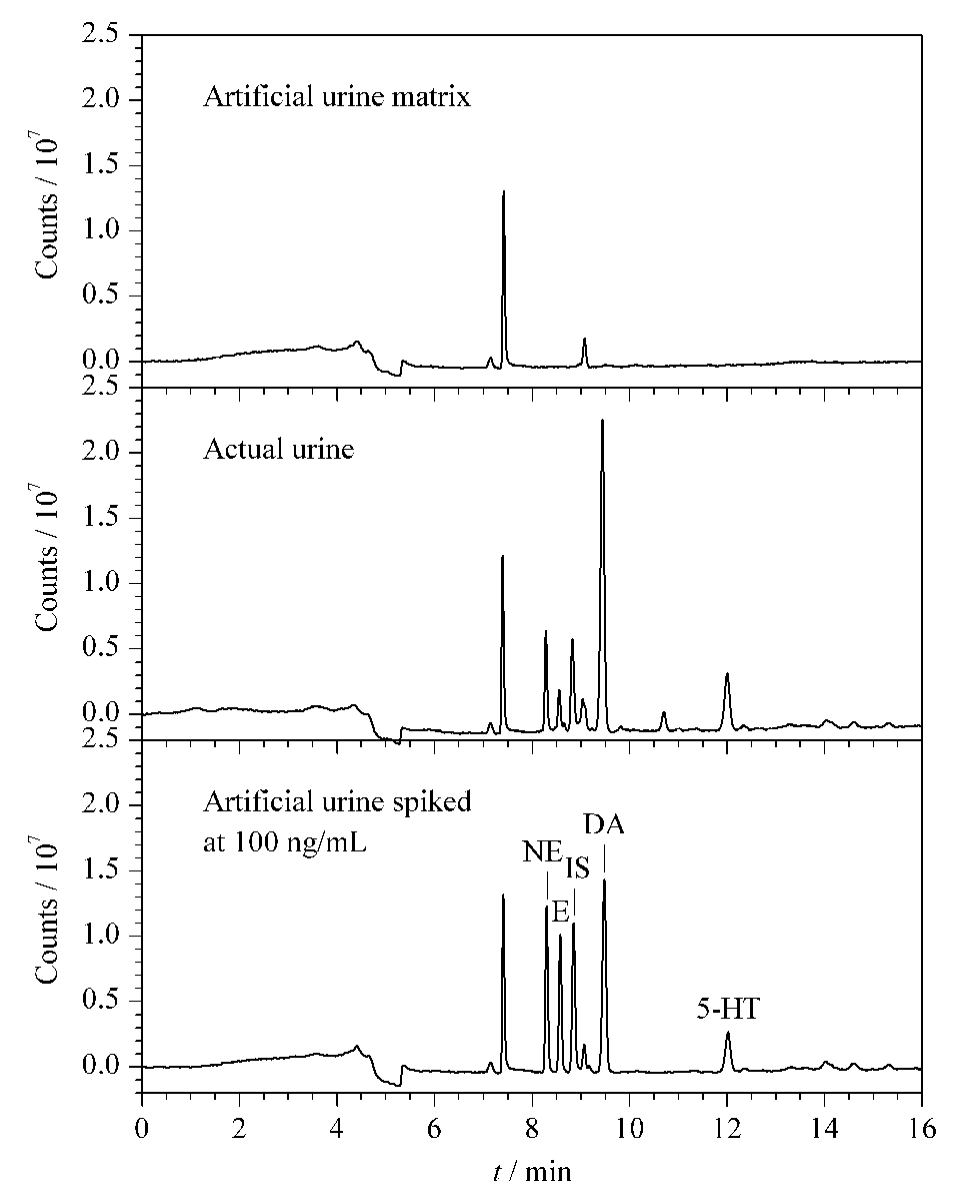
检测实际尿液的色谱图

## 3 结论

本研究采用静电纺丝法制备聚合冠醚复合纳米纤维,将其作为SPE的吸附剂装填固相萃取柱,同时选择双泵柱切换阀系统,设计在线联用程序,将PFSPE与HPLC-FLD进行在线联用。该方法成功应用于人体尿液中NE、E、DA及5-HT的检测,能够有效地富集目标分析物和净化尿液中的内源性杂质,该方法加标回收率高,精密度良好,在1~200 ng/mL范围内有着良好的线性。此外,聚冠醚复合纳米纤维的稳定性较好,10 mg装填量的PFSPE柱可以重复使用近百次,相比离线操作一次使用2~3 mg,使用效率成百倍提高,大大节省物力。

总之,本研究建立的PFSPE-HPLC在线联用方法是一种更简便、有效、环境友好的检测方法。该方法能够集样品前处理与分析检测于一体,分析的自动化程度高,在很大程度上扩展了PFSPE样品前处理技术的应用。此外,该聚冠醚复合纳米纤维可以重复使用,因此可长时间自动运行,实现大批量尿液中NE、E、DA及5-HT的在线自动化富集和分析。该方法展现出很大的临床应用前景,适用于临床尿液样本中NE、E、DA及5-HT的检测,可以为相关疾病提供辅助诊断技术支持。
